# Case Report: Late postpartum eclampsia-associated reversible posterior encephalopathy syndrome with anti-DPPX encephalitis

**DOI:** 10.3389/fimmu.2026.1967757

**Published:** 2026-10-30

**Authors:** Xuanlin Xing, Yuyan He, Liumei Ma, Linming Zhang

**Affiliations:** 1 Department of Emergency, The First Affiliated Hospital of Kunming Medical University, Kunming, Yunnan, China; 2 Department of Neurology, The First Affiliated Hospital of Kunming Medical University, Kunming, Yunnan, China

**Keywords:** autoimmune encephalitis, case report, DPPX, late postpartum eclampsia, posterior reversible encephalopathy syndrome

## Abstract

Posterior reversible encephalopathy syndrome (PRES) is a clinical-radiological syndrome characterized by headaches, seizures, visual disturbances, and focal neurological deficits, accompanied by specific cranial imaging findings. Common triggers of PRES include hypertension/eclampsia, renal disease, autoimmune disorders, and drug abuse. PRES associated with autoimmune encephalitis is a rare phenomenon that presents diagnostic challenges in clinical settings, often leading to missed diagnosis and delayed treatment. We report a case involving a patient with late-onset eclampsia, occurring 26 days after cesarean section, who exhibited dual neurological syndromes of reversible posterior encephalopathy syndrome and anti-DPPX encephalitis. MRI resonance imaging revealed vasogenic cerebral edema and extensive enhancing lesions.

## 1 Introduction

Posterior reversible encephalopathy syndrome (PRES) is a neuroclinical condition characterized by a combination of clinical symptoms and distinct cranial imaging findings. These manifestations may arise from impaired autoregulatory mechanisms in the brain, leading to vasogenic edema resulting from acute or severe fluctuations in blood pressure (1). Various predisposing factors contribute to the development of PRES, such as hypertension, renal disease, certain medications, autoimmune disorders, malignancies, solid organ or stem cell transplantation, and sepsis (2–5). Dipeptidyl peptidase-like protein 6 (DPPX) antibody-mediated encephalitis is a rare subtype of autoimmune encephalitis (AE), characterized by prodromal weight loss, gastrointestinal dysfunction, neuropsychiatric disorders, and the classic triad of central nervous system hyperexcitability.

In this case study, the patient presented with headache, dizziness, and visual disturbances 26 days after the cesarean section. Dysarthria also occurred on the first day of onset, followed by the development of tonic-clonic seizures during hospitalization. Cranial MRI revealed posterior reversible encephalopathy syndrome and meningoencephalitis, with positive DPPX antibodies. Following symptomatic treatment, including intracranial pressure reduction and blood pressure control, the patient’s clinical and imaging features showed improvement. To our knowledge, this is the first documented case of late postpartum eclampsia, characterized by both posterior reversible encephalopathy syndrome and anti-DPPX encephalitis. We aimed to bridge the knowledge gap regarding this condition, enhance physicians’ ability to recognize such rare occurrences, and promote timely and accurate diagnosis and intervention.

## 2 Case report

The patient was a 34-year-old female weighing 84 kg, with a body mass index (BMI) of 32.8 kg/m^2^. She presented with persistent dizziness, headache, blurred vision, and visual distortion for two days prior to her visit. Visual distortion manifests as the perception of individuals with significantly enlarged heads, abnormally large eyes, or the misperception of black hair as white. The patient also experienced dysarthria on the first day of symptom onset, Cranial MRI performed by the emergency department of our hospital revealed the following findings: 1. multiple small punctate abnormal signals in the bilateral periventricular and semioval center areas, suggestive of demyelinating changes; 2. Bilateral occipital white matter lesions, indicative of reversible posterior encephalopathy syndrome; and 3. Linear abnormal signals in parts of the bilateral frontal, parietal, occipital, and temporal lobe sulci, the cerebral longitudinal fissure, the cerebellar vermis, bilateral cerebellar hemispheres, and the dura mater were, suggestive of meningoencephalitis (**Figure 1**).

**Figure 1:**
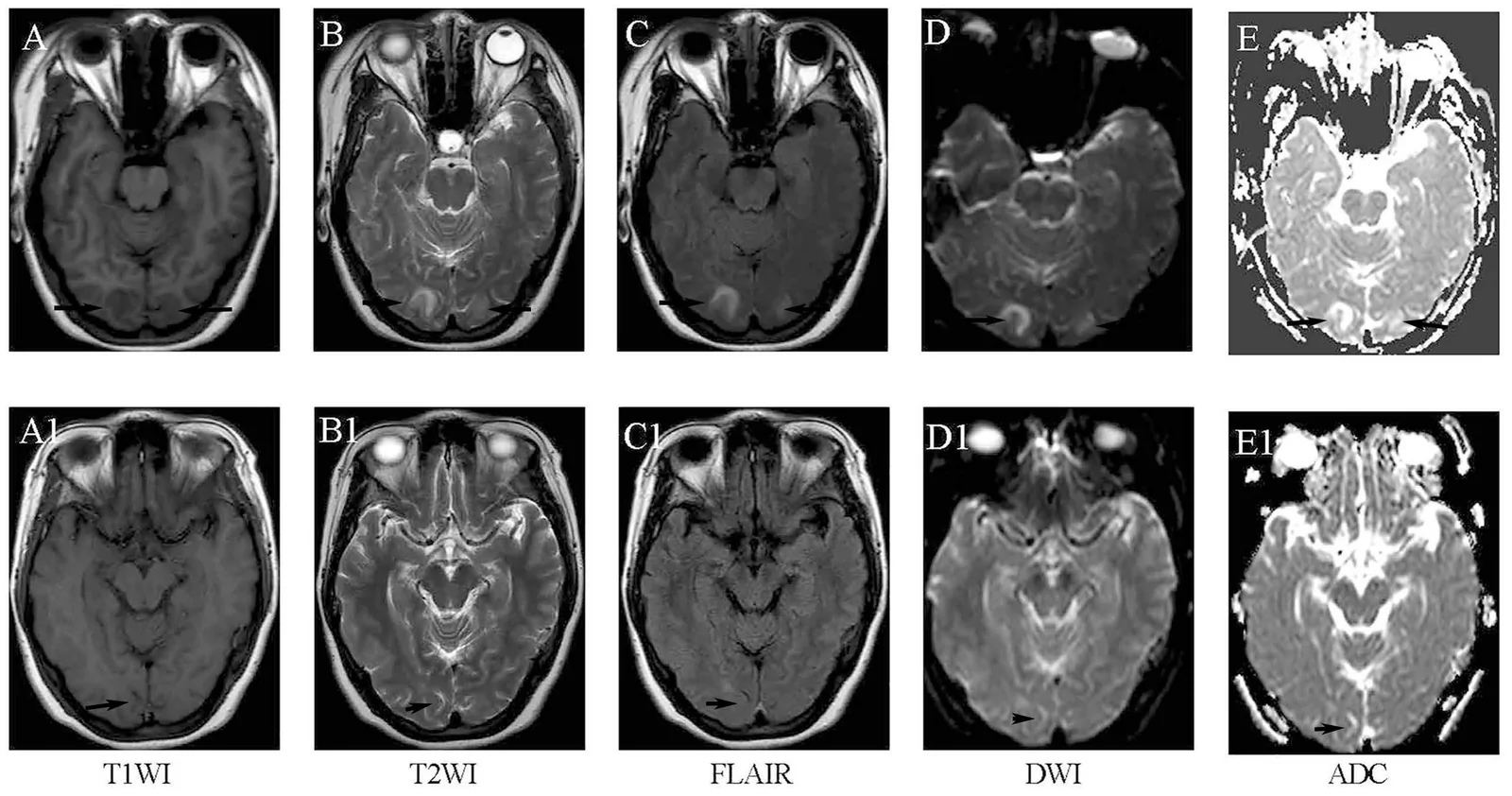
Axial brain MRI scan images of bilateral occipital white matter lesions in different sequences, A1–E1 (July 16, 2021), demonstrate that the vasogenic edema lesions in the occipital lobe have significantly reduced compared to those observed in sequences A–E (July 4, 2021). The sequences A/A1–E/E1 correspond to T1-weighted, T2-weighted, FLAIR, diffusion-weighted imaging (DWI), and apparent diffusion coefficient (ADC), respectively.

The patient’s physical examination revealed a Glasgow Coma Scale score of E4V4M6, a temperature of 36.4 °C, a pulse rate of 64 beats per minute, a respiratory rate of 10 breaths per minute, and a blood pressure reading of 169/82 mmHg. Notably, the patient exhibited intermittent horizontal diplopia in both eyes; however, cardiovascular, pulmonary, abdominal, and neurological examinations were all within normal limits.

The patient reported no history of hypertension, diabetes, thyroid disease, infectious diseases, or psychiatric disorders. Additionally, the patient denied any current medication use and any family history of diseases. Furthermore, the patient does not wear glasses. The patient has given birth to three children and underwent cesarean sections in 2009 and 2013. She subsequently had another cesarean section on June 7, 2021, due to a recurrent scarred uterus.

The examination results indicated the following laboratory values: white blood cell count (WBC) at 8.03 × 10^9/L, neutrophil percentage (NEUT%) at 75.9%, platelet count (Plt) at 101 × 10^9/L, C-reactive protein (CRP) at 16.9 mg/L, procalcitonin (PCT) at 0.18 ng/L, erythrocyte sedimentation rate (ESR) at 33 mm/h, and D-dimer at 1.88 mg/L. Serological tests for syphilis, cytomegalovirus, herpes simplex virus, Epstein-Barr virus, hepatitis B, and HIV, along with evaluations for systemic autoimmune diseases, returned negative results. Chest computed tomography (CT) revealed patchy inflammatory changes in both lungs, while gynecological ultrasonography indicated the presence of residual placenta with partial adhesions. Furthermore, abdominal computed tomography revealed no signs of tumors.

On the second day of hospitalization, the patient experienced a tonic seizure, with a recorded blood pressure of 213/91 mmHg. The seizure was successfully terminated following intravenous administration of midazolam, and blood pressure stabilized at 168/81 mmHg after the patient calmed down. For maintenance therapy, intravenous sodium valproate and oral levetiracetam were continued, with a gradual transition to oral levetiracetam along throughout the course of the illness. On July 7, 2021, cell-based assays (CBA) conducted by Kindstar Globalgene Technology Inc. were utilized to test for antibodies related to paraneoplastic syndrome and autoimmune encephalitis following a lumbar puncture, including anti-NMDA receptor, anti-LGI1, anti-AMPA1/2, anti-CASPR2, anti-GABAA/B, anti-IgLON5, anti-mGluR1/5, anti-DPPX, anti-GlyR1, anti-DR2, anti-GAD65, anti-MOG, anti-Neurexin-3a, anti-Yo, anti-Ri, anti-Caspr2, anti-SOX1, anti-Ma1/2, anti-Amphiphysin, anti-CV2, anti-Recoverin, anti-AQP4and anti-GFAP. The patient’s cerebrospinal fluid (CSF) opening pressure measured 210 mmH2O, and routine and biochemical analyses returned normal results (see **Table 1**). Furthermore, the next-generation sequencing (NGS) analysis of the CSF yielded negative results. Subsequently, on July 10, 2021, we conducted a 15-hour electroencephalogram (EEG) monitoring session for the patient, which revealed no abnormalities. On July 12, 2021, anti-dipeptidyl-peptidase-like protein-6 (DPPX) antibodies were identified as positive in the patient’s serum (titer, 1:10) and cerebrospinal fluid (CSF) (titer, 1:1), with positive results observed in brain tissue sections (TBA), while all other antibodies tested negative. Consequently, the patient was diagnosed with late-onset postpartum eclampsia complicated by posterior reversible encephalopathy syndrome and DPPX antibody-associated encephalitis. However, the patient declined immunotherapy for personal reasons. Subsequently, treatment with mannitol and glycerol fructose was administered to reduce intracranial pressure, alongside blood pressure control and maintenance of fluid and electrolyte balance, which led to symptomatic relief of dizziness, headache, and visual disturbances. On July 16, 2021, brain MRI showed improvement in the lesions (**Figure 2**), after which the patient was discharged against medical advice.

**Table 1:** Measurement results of cerebrospinal fluid in patients.

Patient cerebrospinal fluid testing.		
Content	July 7, 2021.	July 16, 2021.
cerebrospinal fluid white blood cell count. (× 10^6^/L)	1	6
Biochemistry		
AST(U/L)	16.9	21.7
ALT(IU/L)	1.4	0.1
AST/ALT	12.07	217
ADA(U/L)	0.2	0.2
Glu(mmol/L)	3.33	3.96
MTP(g/L)	0.24	0.23
uMA (mg/L0	144	141.3
Cl(mmol/L)	129.3	130.5
CK(IU/L)	1.2	1.3
LDH(IU/L)	24	26
Immunoglobulin		
IgM(mg/L)	0.69	
IgG(mg/L)	25.8	
IgA(mg/L)	2.52	

**Figure 2:**
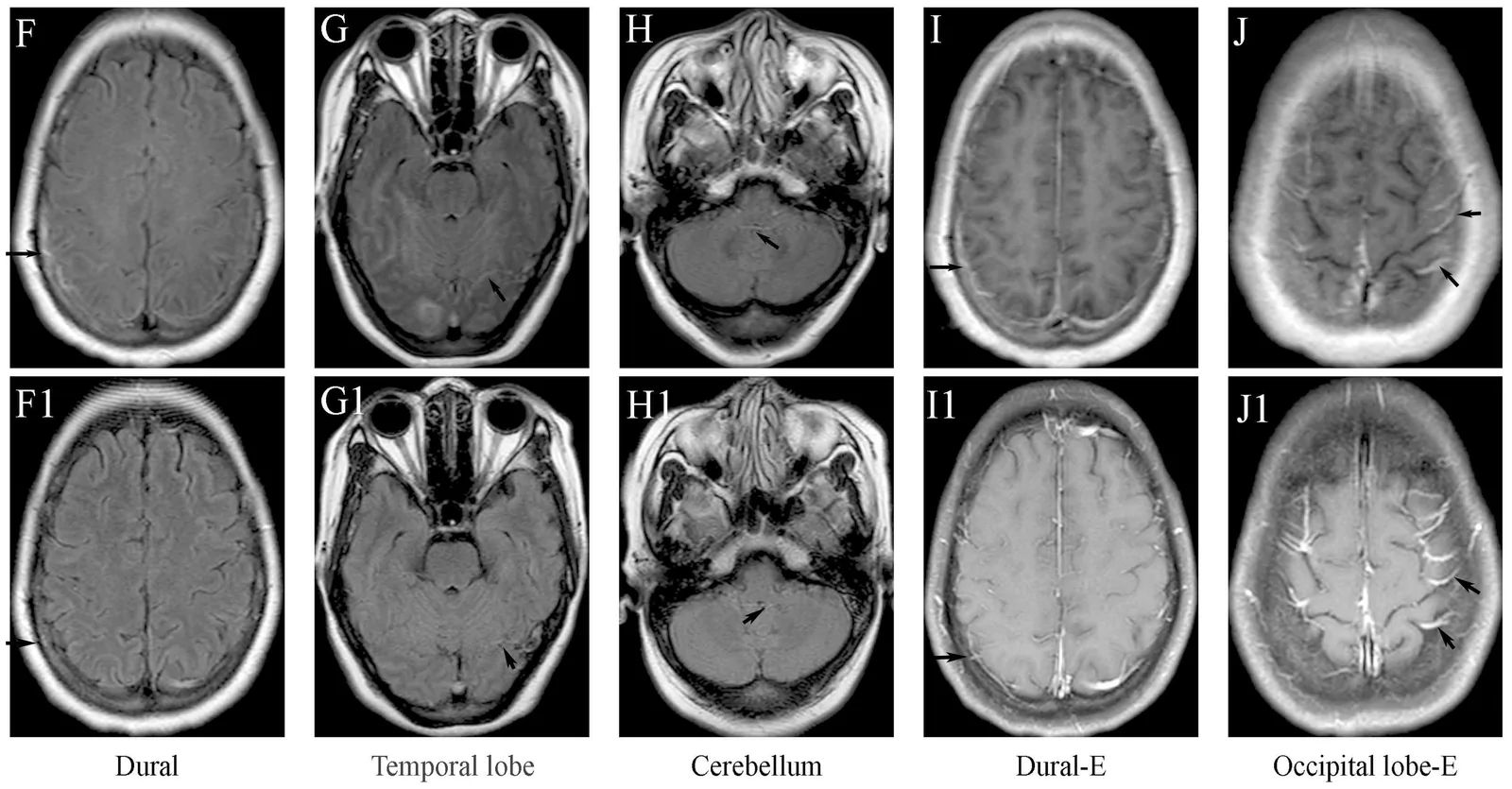
Axial brain MRI scans reveal linear enhancement lesions located at multiple intracranial sites. A comparison of images from July 16, 2021 (F1-J1) indicates a fading of these linear enhancement lesions in contrast to those from July 4, 2021 (F-J). The lesions are observed in F/F1 (dura mater), G/G1 (temporal lobe), and H/H1 (cerebellum), all of which are T2-FLAIR images. Additionally, I/I1 (dura mater) and J/J1 (occipital lobe) are depicted as contrast-enhanced T1-weighted images.

During the follow-up period from discharge until August 25, 2025, the patient did not experience any recurrence or rehospitalization. Although intermittent headache symptoms were reported during this time, blood pressure control remained largely stable, visual impairment had completely resolved, and the modified Rankin Scale (mRS) score was 0, please refer to **Table 2** for the patient's clinical timeline.

**Table 2:** Chronological timeline of clinical events.

June 7, 2021.	July 2, 2021.	July 4, 2021.	July 6, 2021.	July 7, 2021.	July 10, 2021.	July 12, 2021.	July 16, 2021.	August 25, 2025.
Third cesarean section.	The patient presents with symptoms of dizziness, headache, blurred vision, and dysarthria.	The patient’s Glasgow Coma Scale (GCS) score was recorded as E4V4M6. Magnetic Resonance Imaging (MRI) of the brain revealed vasogenic cerebral edema accompanied by extensive enhancing lesions. Nimodipine was administered through continuous intravenous infusion, in conjunction with piperacillin for the treatment of infections.	The patient presented with tonic-clonic seizures, with a recorded blood pressure of 213/91 mmHg. An intravenous infusion of midazolam effectively terminated the seizures. Maintenance therapy was continued with intravenous valproate and oral levetiracetam, while mannitol and glycerol fructose were administered to reduce intracranial pressure.	The patient’s dizziness and headache showed improvement, and their speech was clear, with a Glasgow Coma Scale (GCS) score of E4V5M6. A lumbar puncture was performed to test for paraneoplastic antibodies and autoimmune encephalitis antibodies. Concurrently, the patient’s serum vitamin levels, antinuclear antibody panel, rheumatoid-related antibodies, antiphospholipid antibodies, thyroid function, and serum pathogen tests returned negative results.	The patient’s cerebrospinal fluid next-generation sequencing (NGS) results were negative. Occasional episodes of blurred vision were noted. Valproic acid was discontinued once its blood concentration reached the therapeutic standard, and levetiracetam was subsequently administered orally as the sole agent for maintenance antiepileptic therapy.	Dipeptidyl peptidase-like protein-6 (DPPX) antibodies were found to be positive in the patient’s serum (titer, 1:10) and cerebrospinal fluid (CSF) (titer, 1:1). Additionally, the test for brain tissue section (TBA) was also positive; however, the patient declined immunotherapy due to breastfeeding.	The patient’s brain MRI demonstrated improvement in the lesions, and the patient was subsequently discharged following a repeat lumbar puncture.	During the follow-up period, the patient did not experience any recurrence or readmission. Intermittent headache symptoms occurred occasionally; however, blood pressure control remained generally stable. Visual impairment had completely resolved, and the modified Rankin Scale (mRS) score was 0.

## 3 Discussion

PRES, or Reversible Posterior Leukoencephalopathy Syndrome (RPLS), is a rare neurological disorder that was first described in 1996 at a medical center in England (6). PRES is currently categorized by etiology into three types: infection- related (such as SARS-CoV-2), drug-related (including immunosuppressants, chemotherapeutic agents, cocaine, and ergotamine), and clinical disease-related (encompassing conditions such as hypertension/eclampsia, renal diseases, and autoimmune diseases) (7). Notably, PRES may manifest in the absence of hypertension (8). Characteristic symptoms include sudden onset of headache, seizures, visual disturbances, and MRI findings of white matter changes in the parieto-occipital region. Imaging lesions may also be present in the occipital lobe, frontal lobe, temporal lobe, cerebellum, and brainstem (9). However, the precise pathophysiological mechanisms underlying PRES remain unclear. The current study suggests two potential mechanisms: 1. An increase in arterial blood pressure exceeds the autoregulatory capacity of the brain, resulting in blood-brain barrier dysfunction and subsequent vasogenic edema; 2. Endothelial dysfunction is induced by various endogenous (e.g. renal and autoimmune diseases) or exogenous factors (e.g. chemotherapy and immunosuppressants) (10).

In this case, a 26-day postpartum multiparous woman was admitted with dizziness, headache, and visual disturbances. Brain MRI revealed white matter lesions in the occipital lobe, along with hypertension, which was initially considered Posterior Reversible Encephalopathy Syndrome (PRES). This syndrome frequently manifests during pregnancy and the perinatal period, with a higher incidence in primiparous patients, accounting for 81% of cases. Its association with preeclampsia and eclampsia is well established. Postpartum eclampsia (PPE) refers to eclamptic events occurring between 48 hours and 4 weeks after delivery, and there have been increasing reports of late postpartum eclampsia leading to delayed PRES events (11–13). However, the pathogenesis and prevalence of both these conditions remain unclear. In our case, no infectious pathogens were identified in the patient’s cerebrospinal fluid, which aligns with the findings of most previous studies. We hypothesize that retained placental tissue in the uterine cavity following cesarean section may release a series of inflammatory cytokines, thereby triggering an excessive systemic inflammatory response. This response subsequently leads to the activation and damage of systemic endothelial cells. Activated endothelial cells secrete a significant quantity of inflammatory mediators and vasoconstrictive substances, resulting in cerebral vasogenic edema and an inflammatory immune cascade reaction, which ultimately contributes to the development of Posterior Reversible Encephalopathy Syndrome (PRES) and autoimmune encephalitis. Although the patient demonstrated improvements in imaging and clinical signs following supportive treatments aimed at reducing intracranial pressure and controlling blood pressure, these signs did not completely resolve. This observation supports our initial hypothesis regarding the mechanism of PRES. Nevertheless, it is important to consider that autoimmune encephalitis may also induce the release of pro-inflammatory cytokines, such as interleukin-6, which can activate and damage endothelial cells, leading to cerebrovascular edema and the manifestation of PRES. Consequently, the possibility of DPPX antibody-associated encephalitis secondary to PRES cannot be excluded.

Anti-DPPX autoantibody-mediated autoimmune encephalitis is a rare subtype characterized by the widespread presence of DPPX antibodies in the hippocampus, cerebellum, and myenteric plexus, resulting in multifocal manifestations. Approximately 44.2% of patients present with the classic triad of weight loss, central nervous system hyperactivity, and cognitive deficits (14). Furthermore, recent reports have identified additional symptoms, including limb pain, slurred speech, constipation, and sleep disturbances (15, 16). In this case, the patient had recently undergone a cesarean section, which precluded accurate measurement of weight changes. Nevertheless, the patient exhibited clinical manifestations including slurred speech, constipation, and hyperexcitability of the central nervous system. Cranial MRI revealed extensive enhancing lesions. There was no prior history of HIV or syphilis, and blood and cerebrospinal fluid examinations showed no etiological findings, including bacteria, fungi, viruses, or tuberculosis. Furthermore, cerebrospinal fluid testing for paraneoplastic syndrome and autoimmune encephalitis antibodies revealed a positive result for the DPPX antibody, while all other antibodies tested negative. Additionally, the patient’s serum also tested positive for DPPX antibodies. Based on the diagnostic criteria proposed by Graus et al., we attributed the imaging findings associated with the patient’s meningitis to anti-DPPX encephalitis. The diagnosis of autoimmune encephalitis is contingent upon the identification of neuronal surface antibodies in the serum or cerebrospinal fluid (CSF). A Chinese case series study reported that the median levels of DPPX antibodies in the serum and CSF were 1:32 (ranging from 10 to 1,000) and 1:3.2 (ranging from 1 to 10), respectively, as determined by cell-based assay (CBA). However, DPPX antibodies are typically synthesized intrathecally, suggesting that CSF testing may provide a more reliable assessment than serum testing. In our case, low-titer DPPX antibodies were detected in both the CSF and serum of the patient by CBA. The diagnosis of autoimmune encephalitis is contingent upon the detection of neuronal surface antibodies in the serum or cerebrospinal fluid (CSF). A case series study conducted in China revealed that the median levels of DPPX antibodies in the serum and CSF, as determined by cell-based assay (CBA), were 1:32 (range: 10-1,000) and 1:3.2 (range: 1-10), respectively. However, it is important to note that DPPX antibodies are typically synthesized intrathecally, suggesting that CSF testing may provide more reliable results than serum testing. In our case, low-titer DPPX antibodies were identified in both the patient’s CSF and serum by CBA. In a report by Liu et al. on anti-DPPX encephalitis, the patient’s cranial MRI demonstrated diffuse enhancement, which is atypical for such a widespread distribution of lesions and MRI enhancement, typically occurring only in isolated cases (17). In a multicenter study, approximately 42.9% of patients with anti-DPPX encephalitis exhibited MRI abnormalities, including cortical enhancement, hippocampal swelling or atrophy, and cerebellar atrophy (18). Notably, the brain MRIs of patients with anti-DPPX encephalitis may also appear normal. From the identification of anti-DPPX encephalitis until September 2020, among the 51 reported patients, only nine cases exhibited brain MRI abnormalities, with these abnormalities occurring more frequently in Chinese patients than in non-Chinese patients (14). Therefore, even if brain MRI appears normal, screening for DPPX-AE should still be conducted if the patient presents with typical clinical symptoms.

In 2019, Kim et al. reported a case of LGI1-AE that initially presented as PRES (19). Another case of LGI1-AE from India also exhibited neuroimaging features resembling those of PRES (20). Furthermore, anti-Caspr2 encephalitis can radiologically mimic PRES (21). To our knowledge, this is the first reported case of PRES and DPPX encephalitis, with brain MRI revealing the imaging characteristics of both conditions. PRES may lead to disruption of the blood-brain barrier, and inflammatory mediators released following endothelial cell injury can traverse the compromised blood-brain barrier, exposing neuronal membrane antigen epitopes, and potentially triggering AE. Primary treatment for Posterior Reversible Encephalopathy Syndrome (PRES) involves supportive measures, including addressing triggering factors, maintaining fluid and electrolyte balance, controlling blood pressure, and managing seizures (22). Most cases of PRES are reversible; however, 10%-20% of cases may result in persistent neurological sequelae, with severe instances potentially leading to death (22, 23). Immunotherapy is a critical component of autoimmune encephalitis (AE) treatment, and the initiation of early immunotherapy has been shown to significantly enhance the prognosis of individuals with AE. Previous studies have indicated that anti-DPPX encephalitis has favorable outcomes when treated with combined immunotherapy, with 82.9% of patients demonstrating a good long-term prognosis (defined as greater than 12 months). Nevertheless, some patients continue to experience long-term cognitive deficits (14, 17). Given the rarity of these cases, the number of reported cases remains limited, and research in this area is still in its early stages. Currently, the sample sizes of existing studies are small, and there is a notable lack of large-scale research.

In summary, this case illustrates delayed Posterior Reversible Encephalopathy Syndrome (PRES) secondary to late postpartum eclampsia, and represents the first reported association of PRES with anti-DPPX encephalitis. The causal relationship and mechanisms linking autoimmune encephalitis and PRES remain unclear, highlighting the need for further investigation through additional cases and experimental studies to elucidate the pathogenesis of PRES-AE. It is noteworthy that autoimmune encephalitis can mimic both the radiological and clinical features of PRES. Therefore, when clinical manifestations and prognosis do not align with the typical presentations of PRES, the possibility of autoimmune encephalitis should be promptly considered. We recommend that clinicians maintain vigilance for autoimmune encephalitis during the treatment of PRES to prevent delays in diagnosis and treatment, thus enhancing the clinical outcomes and prognosis.

However, the article has certain limitations. Firstly, the patient did not receive standardized treatment for autoimmune encephalitis, and the absence of continuous antibody testing precluded the assessment of the relationship between imaging changes, clinical improvement, and treatment response. Secondly, this case report, which is presented for the first time, spans multiple medical specialties, thereby complicating the clarification of the causal relationship between anti-DPPX encephalitis, PRES, and late postpartum eclampsia. These limitations impede the evaluation of disease observation and treatment response; however, this case provides valuable support in the literature and underscores the necessity for further investigation.

## 4 Patient perspective

At follow-up, the patient reported that the condition was very dangerous during the early stages of onset, and the coexistence of multiple diseases had led to a loss of confidence. However, the surprisingly good treatment outcome has resulted in a shift in perspective. Now, both the patient and their family members have transitioned from a lack of understanding regarding the disease to actively monitoring routine examinations, such as blood pressure and weight.

## Data Availability

The original contributions presented in the study are included in the article/supplementary material. Further inquiries can be directed to the corresponding authors.
